# Differential Display Analysis of 2,3,7,8-Tetrachlorodibenzo-*p*-dioxin Identified Induction of Ras-related Nuclear Protein Binding Protein2 (RanBP2) Gene

**DOI:** 10.5487/TR.2009.25.1.035

**Published:** 2009-03-01

**Authors:** Donghak Kim, Young-Ran Lim, Hyoung-Goo Park, Beom Joon Kim, Young-Jin Chun

**Affiliations:** 16 Department of Biological Sciences, Konkuk University, Seoul, 143-701 Korea; 26 Department of Dermatology, College of Medicine, Korea; 36 College of Pharmacy, Chung-Ang University, Seoul, 156-756 Korea

**Keywords:** TCDD, Differential display analysis, RanBP2

## Abstract

TCDD (2,3,7,8-tetrachlorodibenzo-*p*-dioxin) and related halogenated aromatic hydrocarbons elicit a diverse spectrum of biochemical and toxic responses in laboratory animals and mammalian cells in culture. Toxicity and carcinogenicity of TCDD is well established but the molecular mechanism is still poorly understood. Here, we found the noble responsive genes to TCDD using the differential display analysis. Treatment of HepG2 cells with TCDD showed a significantly different mRNA expression pattern from the untreated cells in differential display analysis. The differentially displayed bands were isolated and used as probes in dot blot and Northern blot analyses. Of thirty-five isolated differentially displayed bands, only two bands were confirmed as positive in dot blot and Northern blot analyses. The nucleotides sequences of these clones were analyzed and the search of Genebank database revealed that one clone is highly homologous with RanBP2 (Ras-related nuclear protein binding protein2; 92%) and the other is an unknown gene. RanBP2 is a nucleoporin with SUMO E3 ligase activity that functions in both nucleocytoplasmic transport and mitosis and its role as a novel tumor suppressor has been recently proposed. Thus, these results may suggest the clue elucidating the toxic mechanism of TCDD through RanBP2.

## INTRODUCTION

2,3,7,8-Tetrachlorodibenzo-*p*-dioxin (TCDD) is the prototype congener of a large group of halogenated polycyclic hydrocarbon that cause a profusion of apparently unrelated toxic effects. In rodents, TCDD is one of the strongest tumor promoters ever tested in animal model system. It causes an elevated incidence of hepatic carcinoma and pulmonary and skin tumors (Kociba *et al.*, 1978; Mukerjee, 1998) and promotes tumor formation of the classical tumor promoter 12-O-tetradecanoylphorbol-13-acetate (TPA) in the skin of hairless mice (Knutson and Poland, 1982; Kohle *et al.*, 2008; Poland and Knutson, 1982). Because TCDD is not a mutagen, it has been proposed that it promotes neoplastic transformation in cells that have already been initiated (Geiger and Neal, 1981; Haque *et al.*, 2005; Neal *et al.*, 1982), but the molecular basis of this activity has yet to be resolved. In humans, exposure to dioxin and to various other polychlorinated phenol agents causes a long-lasting skin disease characterized by the hyperkeratinized of follicular sebocytes (Suskind, 1985; Zugerman, 1990). In addition, long-term epidemiological studies have established a link between exposure to high doses of TCDD and certain types of cancer and cardiovascular disease (Fingerhut *et al.*, 1991; Flesch-Janys *et al.*, 1995; Manz *et al.*, 1991).

TCDD has been used as a model compound for investigating the cellular and molecular mechanisms of aryl hydrocarbon (Gasiewicz *et al.*, 2008; Poland and Knutson, 1982; Raunio and Pelkonen, 1983). The major mechanism of action of these compounds is to activate the Ah receptor (AhR) to a form that binds to specific gene regulatory sequence elements, called xenobiotics responsive elements (XREs), through heterodimerization with the AhR nuclear translocator protein (Arnt) (Reyes *et al.*, 1992). The Ah gene battery comprises at least six genes: two Phase I genes, CYP1A1 and CYP1A2; and four Phase II genes, Nmo-1, Aldh-1, Ugt-1, and Gt-1. All six genes appear to be regulated positively by TCDD and other ligands of the Ah receptor (Nebert *et al.*, 1990).

The mRNA differential display technique is a powerful tool for identifying and cloning differentially expressed genes (Liang and Pardee, 1992; Stein and Liang, 2002). In original method, mRNA is first reverse-transcribed into cDNA with 3'-oligo-dT primers anchored to the beginning of the poly(A) tail. Then, cDNA is amplified in a PCR mixture containing the 3'-anchor primer, the 5'-arbitrary primers, and radioactive deoxyribonucleoside triphosphates. The advantage of mRNA differential display lies in its sensitivity and simplicity. It has been used in systematic searching for genes that are differentially expressed (Stein and Liang, 2002).

In this study, the differential display approach was applied to study the molecular mechanism of TCDD toxicity and carcinogenicity. Several TCDD responsive genes have shown the altered pattern of gene expression and the control of RanBP2 gene expression related to TCDD suggested a clue to resolve the molecular basis of its toxicity.

## MATERIALS AND METHODS

***Cell culture.*** HepG2 cell line (human hepatoma) was from Korea Research Institute of Bioscience and Biotechnology (Daejon, Korea). Cells were grown in Dulbecco's Modified Eagle Medium (DMEM, Life Technologies, Grand Island, NY) supplemented with 10% FBS, 100 units/ml penicillin and 100 μg/ml streptomycin. After reaching confluence, the cells were treated with 10 nM TCDD (in DMSO).

***RNA Isolation.*** Cells were harvested 24 h after TCDD treatment, and total RNA was isolated using Tri Reagent (Molecular Research Center, Cincinnati, OH). Briefly, cell pellets were suspended in 1 ml Tri solution and then vigorously vortexed with the addition of 200 μl chloroform. After standing 5 min at room temperature, the suspensions were centrifuged at 10,000 g for 10 min. The aqueous upper phase was transferred to a new tube, mixed with equal volume of isopropanol, and then placed at room temperature for 10 min to precipitate RNA. Sedimentation at 1,000 g for 10 min was again performed and the resulting pellet was rinsed with 0.5 ml of 70% ethanol. After vacuum dried, the pellets were dissolved in nuclease-free water (Promega, Madison, WI). The RNA solution was boiled for 5 min at 70°C and quick chilled in ice to remove secondary structures, which may interfere following reverse transcription reaction. The concentration of total RNA was measured at $OD_{260/280}$, and the integrity of RNA was confirmed by 1% agarose gel electrophoresis.

***Differential display analysis.*** Reverse transcription reactions were done with different one-base-anchored HT11M primers (where M may be G, A, or C) according to the instructions (GenHunter Corporation, Nashville, TN). A 19 μl reverse transcription reaction mixture containing 2 μl (0.1 μg/μl) total mRNA, 4.0 μl 5 × RT buffer (125 mM Tris-Cl, pH 8.3, 188 mM KCl, 7.5 mM $MgCl_2$, and 25 mM DTT), 1.6 μl dNTP (250 μM), 2.0 μl HT11M (2 μM) and 9.4 μl $H_2O$ was incubated in programmable thermocycler (Applied Biosystems, Foster City, CA) (65°C, 5 m → 37°C, 60 m → 75°C, 5 m). After 10 min at 37°C, 1 μl AMV reverse transcriptase was added to each tube, and quickly mixed well and the incubation was continued. The 75°C incubation period was intended to inactivate the reverse transcriptase without denaturing the mRNA/cDNA duplex to prevent initial mispriming by the arbitrary primer in PCR reactions.

Deoxyoligonucleotides with arbitrary sequences (GenHunter Corporation) were used as upstream primers and the RT primers (used in reverse transcription reactions) were used as downstream primers. The total 20 μl reaction mixture contained 0.2 μM upstream primer, 0.2 μM downstream primer, 1.6 μl dNTP (25 μM), 2 μl RT-mix from reverse transcription reaction, 2 μl 10 × PCR buffer, 1 μl α-[$^{35}$S] dATP (1200 Ci/mmole, Amersham, Arlington, IL) and 1 unit *Taq* DNA polymerase. After adding 25 μl mineral oil, amplification was done using programmable thermocycler with 40 cycles by denaturation at 94°C for 30 s, annealing at 40°C for 2 min and extension at 72°C for 30 s and additional 5 min extension at 72°C. After preparing 6% denaturing polyacrylamide gel in TBE buffer, the gel was pre-run for 30 min. The urea in the wells was flushed before loading samples. 3.5 μl of each sample was mixed with 2 μl of loading dye (95% formamide, 10 mM EDTA, pH 8.0, 0.09% xylene cyanole FF and 0.09% bromophenol blue) and incubated at 80°C for 2 min and immediately loaded on wells. After electrophoresis at 1700 V, the gel was dried under vacuum on gel drier at 80°C for 1 h, and then exposed to the X-ray film for 3 days.

The differentially displayed bands were excised and the gel slices were soaked in 100 μl $H_2O$ for 10 min and were boiled for 15 min. After spinning for 2 m, the supernatant was transferred to a new tube and precipitated with 10 μl of 3 M sodium acetate, 5 μl of glycogen (10 mg/ml) and 450 μl of 100% ethanol in -80°C freezer. After spinning for 10 min at 4°C, the supernatant was removed and the pellet was rinsed with 200 μl ice-cold 85% ethanol. The pellet was dissolved with 10 μl $H_2O$ and 4 μl of this sample were used for reamplification. The reaction was carried out in 40 μl solution using identical conditions to the amplification procedure. After the re-amplification, 10 μl of each sample was run on a 1.5% agarose gel and stained with ethidium bromide.

***Dot blot and northern blot analysis.*** For dot blot experiments, the dot blot kit (Bio-Rad, Hercules, CA) was used with various amounts of mRNA samples. Designed amounts of total mRNA were blotted onto nylon membrane filters (Amersham) and the filters were cross-linked using the UV Stratalinker 1800 (Stratagene, La Jolla, CA). After drying, the filters were prehybridized for 2 h in a prehybridization buffer containing 50% formamide, 10 μg/ml of denatured salmon sperm DNA, 5 × SSC, 0.2% SDS, and 5 × Denhart's solution (0.02% Ficoll, 0.02% polyvinyl pyrrolidone 360, and 0.02% BSA) at 42°C. After prehybridization, the filters were hybridized with [$^{32}$P]-labeled probes overnight. Nylon membrane filters were washed with 3 changes of 4 × SSC containing 0.1% SDS at 37°C and then dried in air and exposed to an X-ray film at -70°C using an intensifying screen.

***Cloning and DNA sequencing.*** The oligomers of the candidate clones were cloned into pBluescript SK plasmid using *Sma*I restriction enzyme site. The nucleotide sequences of the cloned DNA were analyzed and DNA sequences of the clones were compared using the BLAST Basic Local Alignment Search Tool provided by NCBI (http://blast.ncbi.nlm.nih.gov/Blast.cgi).

## RESULTS

***Differential display.*** HepG2 cells were treated with 10 nM TCDD or with DMSO (control) for 24 h. Total mRNA samples were analyzed using the differential display (DD) method. Three different pools of cDNAs were generated for each sample by reverse transcription with the $T_{12}$HM oligonucleotides and then the cDNA pools were subjected to PCR in combination with 8 arbitrary sequence primers (24 combinations). Electrophoresis was done and the comparison of DD patterns of DMSO treated versus TCDD-treated samples showed 35 bands corresponding to mRNAs of which the expression were altered in response to TCDD (Fig. 1). The individual

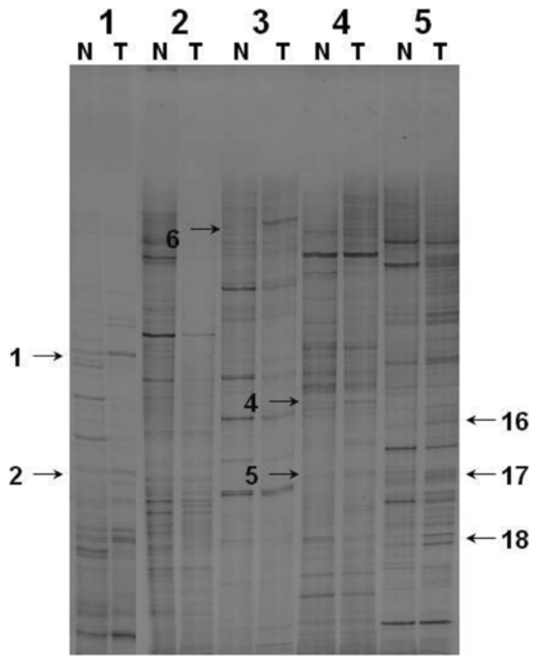

**Fig. 1.** Gel analysis of differentially displayed mRNAs. HepG2 cells were treated with 10 nM TCDD and mRNA was reverse-transcribed using a set of DD primers. The reaction was run on a 6% DNA sequencing gel (N: DMSO-treated; T: TCDD-treated; the arrows represent the selected clones).

bands were cut out and re-amplified by PCR using same combination of primers as in the DD analysis and used as probes in Northern analysis and for DNA sequencing. 10 μl of PCR sample were run on a 1.5%

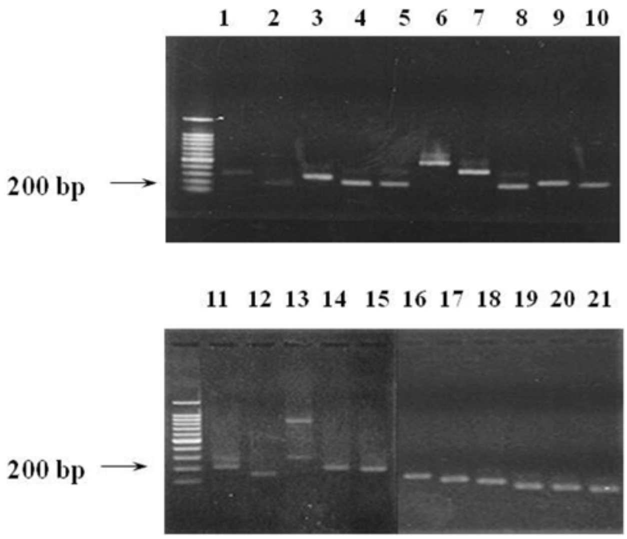

**Fig. 2.** Thirty-five clones selected from differential display analysis. The differentially displayed bands were excised and re-amplified.

agarose gel and stained with ethidium bromide. Most clones were within 100 bp and 500 bp (Fig. 2).

***Gene expression analysis.*** Of thirty-five isolated differentially displayed bands, only two bands were positive in dot blotting assay (Fig. 3A). TCDD significantly induced the mRNAs of these two clones in a concentration-dependent manner. The amounts of mRNAs detected by the specific clone 4 probe were increased with the proportion to the amounts of total mRNAs. The quantification of result showed that the increase level was 3 folds in 4 μg, 8 folds in 8 μg, or 11 folds in 16 μg RNA (Fig. 3B).

To confirm the results by Northern blot analysis, HepG2 cells were treated with DMSO, 0.01 nM, 0.1 nM, and 1 nM TCDD for 24 h. Northern blot analyses were performed with the clone 4 probe and TCDD induced the gene detected by the clone 4 probe in a concentration-dependent manner from 0.01 nM to 1 nM (Fig. 4A). The level of clone 4 mRNA was enhanced to 3.5-fold in 0.01 nM TCDD (lane 2), 4-fold in 0.1 nM TCDD (lane 3), and 7-fold in 1 nM TCDD (lane 4), compared with control (lane1) (Fig. 4B).

***Sequence analysis.*** The nucleotides sequences of two positive clones in mRNA analysis were analyzed

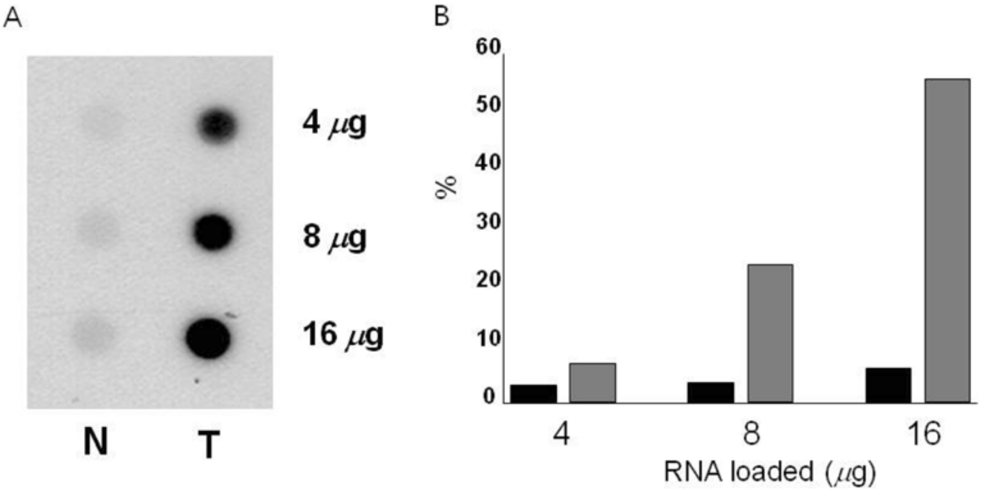

**Fig. 3.** Dot blot analysis. A. HepG2 cells were treated with 10 nM TCDD and 4, 8, 16 μg of RNA were loaded. The blot was probed with [$^{32}$P]-labeled differentially displayed DNA (N: DMSO-treated; T: 10 nM TCDD-treated). B. The quantification of dot blots (solid: DMSO-treated: gray; 10 nM TCDD-treated).

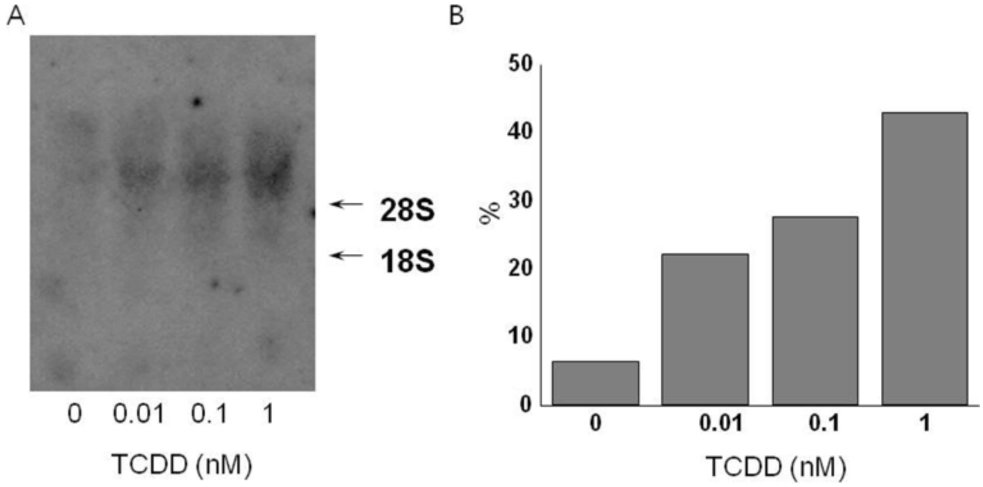

**Fig. 4.** Northern blot analysis. A. Total mRNAs from HepG2 cells were run on a 1.2% agarose-formaldehyde gel and transferred onto a nylon membrane. The blot was probed with [$^{32}$P]-labeled clone-4 DNA probe. B. Induction of clone-4 mRNA expression by TCDD. The Northern blot bands were quantified using gel scanner.

```
 1   TTTTTAAAGT GATGGAAATA
21   ACTTAAATTT TTTTTTCAGT
41   GCACATGTTG CTAAACTTAA
61   GTCGTGGCTA ATCAAGCTTA
81   AGTCGTGG

clone4:  36 TCAGTGCACATGTTGCTAAACTTAAGTCGTGGCTAATCAAG 76
            |||| |||||||||||||||||||||||||||| || ||||
RanBP2:1186 TCAGGGCACATGTTGCTAAACTTAAGTCGTGGC-AAGCAAG 1225
```

**Fig. 5.** BLAST search with clone-4 DNA sequence. The clone-4 DNA was sequenced and BLAST searches with identified DNA sequence revealed RanBP2 gene as a homologous gene. Aligned regions are indicated with *line* below the sequence of clone-4.

with forward and reverse M13/pUC universal primers and the analyzed nucleotides sequences were compared to previously reported sequences using Genebank database. One clone (clone 4) contained a highly homologous region with RanBP2 (Ras-related nuclear protein binding protein2; 92%) and the other was an unknown gene (Fig. 5).

## DISCUSSION

Differential display is a very useful technique to identify the regulation genes. This technique has substantial advantages over early techniques (*e.g.*, substrate libraries or hybridization) since it does not have such a strong bias toward highly abundant genes and is much more versatile, enabling cloning of down-regulated as well as up-regulated genes and allowing a rapid comparison between multiple samples. However, a major problem with this technique is the occurrence of large number of false positives, contamination, and inefficient probes for detection on Northern blotting analysis (Liang *et al.*, 1993). Hence, there are some strategies suggested to identify more promising clones rapidly and efficiently. Vogeli-Lange *et al.* recommended to pool multiple reaction mixtures of previous differential display reactions for use as a probe (Vogeli-Lange *et al.*, 1996) and Kester *et al.* performed fresh differential display reactions and used $^{32}$P instead of $^{33}$P as label in dot blot procedure, which gives a stronger signal and circumvents the need to pool reactions (Kester *et al.*, 1997). Despite competition from newer technologies such as DNA microarrays, differential display has continued to be still commonly used technology for differential gene expression because of the fundamental differences (Liang, 2006). The differential display visualizes the mRNAs in subsets directly without any data normalization after their amplification and labeling by either isotopes or fluorescent dyes. In contrast, DNA microarrays visualize the mRNAs indirectly after the hybridization of extremely complex mRNA probes as first-strand cDNAs with fluorescent labels to a set of cDNA templates spotted on a glass surface. In fact, a cDNA probe used for microarray can be so complex that it consists of as many as 10,000 different species ranging from only a few copies to thousands of copies per cell. Further compounding the problem in signal specificity has been the fact that eukaryotic genes often come in families with many conserved sequences among the family members (Liang 2006; Cho *et al.*, 2001).

In this study, we used the differential display approach to identify new genes that may be a target for the regulation by TCDD. For this purpose, thirty-five differentially expressed clones were isolated and two among them were identified as TCDD-responsive genes measured by the dot blot and the northern blot analyses. Using the BLAST program to search Genebank database, one clone was identified to contain a highly homologous region with RanBP2; whereas the other clone was still not found in Genebank database. RanBP2 is a remarkably large (approximately 350 kDa) protein that contains a leucine-rich region, four potential Ran binding sites and eight zinc finger motif (Navarro and Bachant, 2008). This protein has a role in the signal pathway of Ran through nucleoporin. It up-regulates RanGTPase activity and suppresses GTP dissociation to enhances the transport process of proteins and mRNAs through nuclear pore complex (Yokoyama *et al.*, 1995). Recently, it was reported that RanBP2 contains an unusual SUMO E3 ligase domain as enzymatic function (Pichler *et al.*, 2002) and it has an important role in tumor suppression (Dawlaty *et al.*, 2008). These studies suggested that the increased activity of RanBP2 may change the growth modulation and its induction (or of a highly homologous gene) with RanBP2 by TCDD may be early molecular events surrounding TCDD-induced tumor promotion. Therefore, the delineation of molecular events independent of formation of AhRÞARNT complexes may illustrate a critical clue for an understanding of the mechanisms of TCDD toxicity.

So far, the genes and the mechanism involved in TCDD toxicity are poorly understood. However, the application of differential display method could be useful in the discovery of other unknown genes important for TCDD toxicity. Also, the unknown TCDD responsive gene identified in this study could provide the additional understanding of TCDD-induced toxic or biological responses. Its characterization and physiological role are currently under investigation.

## ACKNOWLEDGMENTS

This study is supported by a grant of the Korea Healthcare technology R&D Project, Ministry of Health & Welfare (A080065).
